# Stents and Stent Mimickers in Endovascular Management of Wide-neck Intracranial Aneurysms

**DOI:** 10.7759/cureus.3420

**Published:** 2018-10-05

**Authors:** Yasir J Khattak, Ayman A Sibaie, Muhammad Anwar, Raza Sayani

**Affiliations:** 1 Radiology, Rashid Hospital, Dubai, ARE; 2 Radiology, The Aga Khan University, Karachi, PAK

**Keywords:** endovascular, stent assisted, wide neck aneurysm, flow diversion, stent-assisted coiling, endovascular coiling, endovascular coiling, coiling

## Abstract

Subarachnoid hemorrhage due to a ruptured cerebral aneurysm is a disastrous event accounting for approximately 5%-15% of all stroke cases and has a high mortality rate. One of the major goals in the management of these patients is to prevent rebleeding by securing the aneurysm either surgically or by endovascular means. Endovascular treatment is considered the first line of treatment for intracranial aneurysms; however, wide-neck aneurysms (WNAs) are specifically difficult to treat by endovascular means due to the difficulty in achieving a stable coil mass inside the aneurysm sac. To overcome this problem, assisted endovascular treatment techniques and devices have evolved over the years. Amongst these, stent-assisted coiling (SAC) techniques provide a scaffold for coil embolization. The concept of the stent-assisted technique inspired creative pioneers to invent new tools like the PulseRider (Pulsar Vascular, Inc. CA, USA) and the pCONUS (Phenox GmbH, Germany), which are a great help in managing wide-neck and bifurcation aneurysms. The concept of stent within stents and its related hemodynamic effect has led to the novel development of flow diverters for reconstructing the arterial wall and correcting the hemodynamic disturbances. In this article, we review the stents and stent-like devices currently in practice for the endovascular management of wide-neck and branch intracranial aneurysms.

## Introduction and background

Following the publication of the ISAT (International Subarachnoid Aneurysm Trial) in 2002 and the ISUIA (International Study of Unruptured Intracranial Aneurysms) in 2003, endovascular means have become the management of first-choice for intracranial aneurysms [1 ,2].

The endovascular treatment of intracranial aneurysms has evolved over the years both in regard to neurointerventional skills as well as the advent of advanced techniques and innovative devices to render the treatment more effective and durable. In 1988 Hilal proposed the use of platinum microcoils; however, coil malpositioning and normal pial artery occlusion were major risks using this method [3, 4]. Most of the drawbacks associated with the Hilal microcoils were overcome by the development of the Guglielmi detachable coils (GDC) in 1991, which were electrolytically detachable [5]. However, the advent of GDC coils did not abolish the challenges related to wide-neck aneurysms (WNAs). The advent of GDC coils paved the way for the development of improvement in coil technology and the addition of 3D coils, bioactive, hydrogel, and TriSpan coils (Boston Scientific, MA, USA ) to the armamentarium [6].

New techniques like balloon remodeling and stent-assisted coiling (SAC) were introduced. In addition, some operators employed the Onyx Liquid Embolic System (Medtronic, MN, USA) to occlude aneurysms unsuitable for coiling or for recurrent aneurysms. Irrespective of these developments, the management of WNAs is complex because of difficulties associated with stabilizing the coils in the aneurysm sac, risk of coil loop protrusion or thrombus dislodgement into the parent vessel, and inadvertent occlusion of side branches. Moreover, branches are frequently seen arising from the aneurysm neck making endovascular treatment technically more arduous [7].

Most new devices target reconstruction of the aneurysm neck, the parent artery, or both. A stable closure of the aneurysm can thus be achieved with lesser recanalization rate in the long term, resulting in a reduced need for repeat surgery and an overall lower risk of rebleeding.

The objective of this article is to present a review of stent-assisted coiling and stent-like devices, which are currently in practice for endovascular management of wide-neck and branch intracranial aneurysms, by employing case examples of patients treated at Rashid hospital, Dubai.

## Review

Remodeling techniques

Amongst the various limiting factors in endovascular treatment of intracranial aneurysms, two of the most important factors to be considered are the aneurysm shape and the width of the aneurysm neck [8]. Aneurysms with a neck diameter greater than 4 mm and a dome-to-neck ratio less than 1:2 are termed wide-neck aneurysms. There is a high risk of coil migration or protrusion into the parent artery if contemporary endovascular techniques are used in these cases. To circumvent this problem, remodeling techniques with balloons and stents were devised. Better results with endovascular treatment in such cases can be achieved if assisted techniques are employed [9].

Stent-assisted coiling (SAC)

One of the commonly employed techniques for the treatment of wide-neck aneurysms is the combined use of coils and stents.

**Figure 1:**
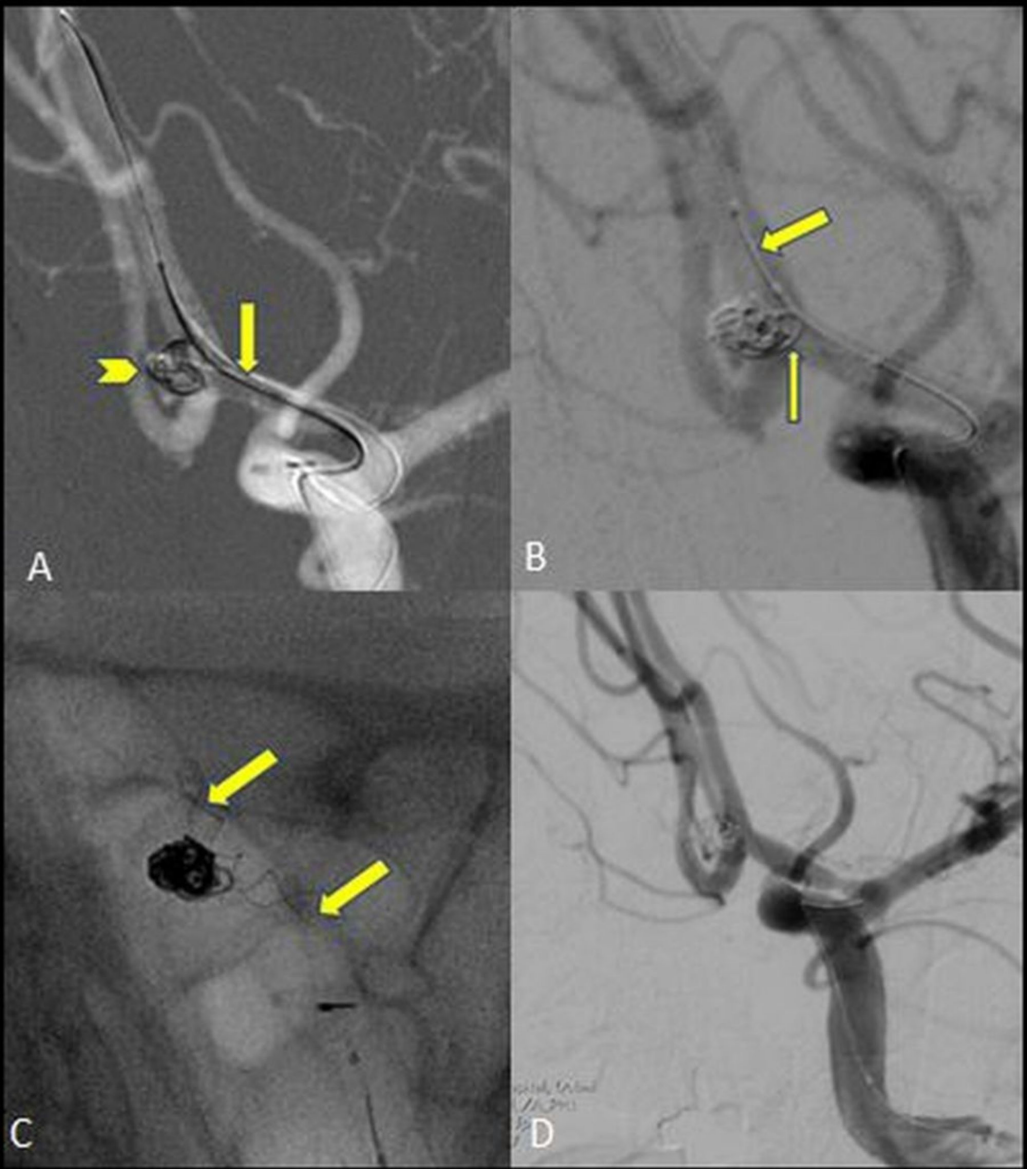
Balloon and stent-assisted coiling for anterior communicating artery aneurism (AcomA) A: Balloon remodeling performed using a double lumen occlusion balloon (Eclipse 2L, Balt Extrusion, Montmorency, France) and coiling done using an Echelon 10 microcatheter (Microvention, CA, USA) with an Avigo 0.14 wire. Coiling of anterior communicating artery aneurysm with balloon protection. B: The balloon is deflated (thick arrow) to assess coil mass movement. A loop of coil (thin arrow) can be seen protruding into the parent vessel after balloon deflation. C: Non-subtracted image showing a 2.5 mm × 18 mm Leo Baby Stent (Balt Extrusion, Montmorency, France) deployed (marked by arrows) across the aneurysm neck compressing the prolapsed coil against the arterial wall maintaining vessel patency. D: Post coiling and stent placement angiogram shows exclusion of aneurysm and patent parent artery.

On many occasions, after balloon deflation a change in coil morphology or protrusion into the parent vessel is evidenced and in such cases a stent is required to maintain the coils in the aneurysm sac. Coiling is performed mostly after placing the stent. Access into the aneurysm sac is achieved using a microwire through the stent struts. Once the wire is progressed into the sac, it is followed by a microcatheter, and coiling is performed subsequently. The permanent placement of stents across the aneurysm neck jails the coils in the sac, which allows dense coil packing and stable neck support, thus preventing delayed compaction and neck recanalization. The presence of the stent also redirects the blood flow while acting as a scaffold and thus contributes to re-endothelialization of the parent artery (Figure 1).

Higashida and colleagues were amongst the first ones who attempted the use of a coronary stent for treating a fusiform aneurysm. They reported placement of a stent across the aneurysm with deployment of coils through the stent’s walls to thrombose the aneurysm outside the stent, thus maintaining a patent lumen [10]. Lanzino and Sekhon in 1998 further expanded upon this concept by reporting five more cases [11, 12].

The initial stents were stiff and thus difficult to navigate through tortuous intracranial vasculature even by experienced hands. Hence these first-generation stents were associated with thrombotic and hemorrhagic complications. Nowadays, with significant improvements in stent design and technology, neurointerventionalists have much more flexible devices available at hand that are easier to navigate even in curved vasculature, providing a safer alternative and thus expanding the indications for their use in hostile anatomy [13].

The commonly used intracranial stents are self-expandable. Some of the common stents available today are the following:

Solitaire® (Ev3 Neurovascular, Inc., Irvine, California, USA)

Leo Plus® (Balt Extrusion, Montmorency, France)

Enterprise® (Cordis Neurovascular, Miami, Florida, USA)

Neuroform EZ ® (Stryker)

LVIS and LVIS Jr (Microvention, Tustin, California, USA)

LEO and LEO+ Baby (Balt, Montmorency, France)

ACCLINO (Acandis, Pforzheim, Germany)

Balloon expandable stents have also been used for intracranial aneurysm treatment [14].

**Figure 2:**
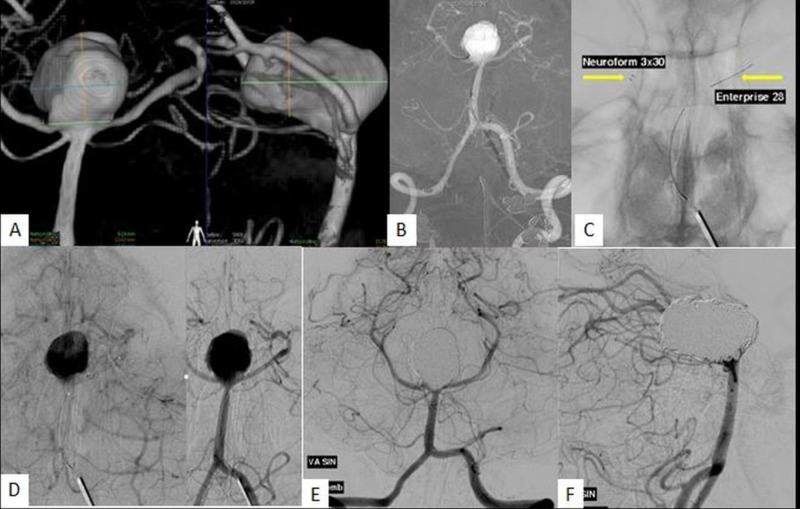
Y-stenting technique for the treatment of a wide-neck aneurysm located at the tip of the basilar artery A-B: Vertebral angiogram showing a large lobulated irregular shaped basilar tip aneurysm with both P1 segments of the posterior cerebral artery (PCA) incorporated within the aneurysm sac. C-D: Arrows pointing to stents placed in bilateral P1 segments of PCAs. The Y-stenting was performed using an Enterprise stent (Codman Neuro, MA, USA) on the right and a Neuroform stent (Stryker, MI, USA) on the left. Catheterization of the aneurysm was performed through the struts of the stents (not shown). Good visualization of the Neuroform markers (arrow in C) is observed. E-F: Post embolization anterior-posterior (AP) and lateral (LAT) angiograms of the basilar artery show exclusion of remnant at the neck.

Stents can be either used alone or in combination with a second stent in a Y-shaped configuration depending on the anatomical location of the aneurysm. If a bifurcation aneurysm neck is off-centered to one side branch, a single stent would usually suffice for treatment; however, if the sac is exactly centered at the bifurcation, the Y pattern of stent deployment can be employed to protect the branches. The technique is well-explained for basilar tip aneurysms and can also be used for wide-neck bifurcation aneurysms at other intracranial locations like middle and anterior cerebral arteries (Figure 2) [15].

One of the limitations of stent-assisted coiling is in cases of ruptured aneurysms. For stent-assisted coiling, the patient needs to be preloaded with anti-platelets to prevent stent thrombosis. In the setting of a subarachnoid hemorrhage, an antiplatelet regime is usually not feasible to follow due to the risk of catastrophic rebleed or inability to perform surgery if the need arises. Although different series show acceptable results of stent-assisted coiling in the setting of subarachnoid hemorrhage [9], we in our center keep stent-assisted coiling as a secondary option for cases with recent subarachnoid hemorrhage.

Although various studies comprising retrospective series have reported heterogeneous complication rates, stent-assisted coiling remains a well-established and useful technique for endovascular management of wide-neck aneurysms [16-18].

In certain situations, alternate techniques are required to safeguard the side branches or branches incorporated in the aneurysm sac. One such case is an obtusely-oriented side branch or a very wide neck. New techniques and novel devices have been devised for such complex cases.

Waffle cone technique

In cases where the side branches arise at an acute angle near the aneurysm neck, posing a very high risk of post embolization compromise, alternate methods of coiling should be employed. Horowitz et al. in 2006 first described one of such method and termed it “waffle-cone” technique because of the typical appearance of the stent-coil combination after treatment [19].

This technique is especially useful for basilar tip wide-neck aneurysms where it is difficult to cannulate bilateral posterior cerebral arteries on account of their location in relation to the aneurysm neck; however, it can also be used for bifurcation aneurysms of anterior communicating and middle cerebral arteries.

**Figure 3:**
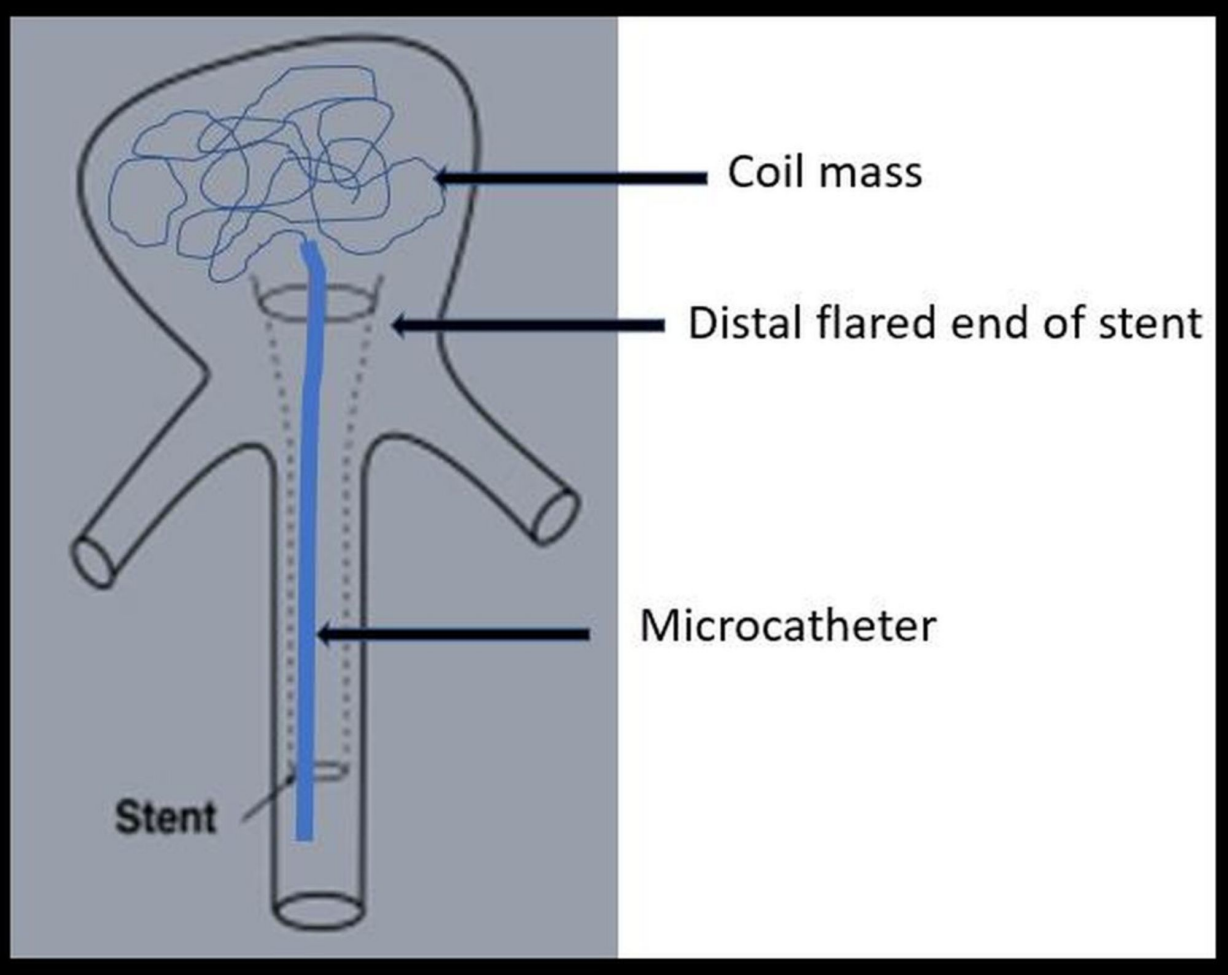
Waffle cone technique Schematic diagram showing stent deployed in the aneurysm neck to provide support. The proximal part of the stent remains in the parent vessel, while coiling is performed through the flared distal end.

In the waffle cone technique, a stent is deployed in the aneurysm neck to provide support. The proximal part of the stent remains in the parent vessel, while coiling is performed through the flared distal end (Figure 3) [20].

The technique thus allows preservation of the parent artery and the side branches. In cases where there is a discrepancy of diameter between the proximal and the distal vessels, stent placement is challenging and risky. The waffle cone technique is advantageous in such situations and provides a better alternative than the conventional stent-assisted coiling approach.

Both open and closed cell design stents have been used for this purpose, each having their own advantage and disadvantages, and neither has been proven to be superior to the other [19, 20].

Solitaire stents that have a closed cell design can be retrieved after full deployment. Moreover, these do not need distal access platinum wires, which are required to be positioned in a distal vessel as is the case with open cell design.

The technique has not been evaluated in larger series and hence its safety isn’t fully assessed. Conventional stent-assisted coiling should thus be the technique of first choice in wide-neck aneurysms. In cases where the conventional technique isn’t feasible, the waffle cone technique provides a useful alternative.

Based on the idea of the waffle cone technique new devices have been developed, including the pCONus (Phenox GmbH, Bochum, Germany) and the PulseRider (Pulsar Vascular, California, USA), which are purpose-built for wide-neck bifurcation aneurysms.

pCONus

The stents used as waffle cones were not purpose-built for that specific procedure and were far from ideal. Thanks to the biomedical advancements in the last decade, amongst many other devices, pCONus (Phenox, Bochum, Germany) was designed to serve as a dedicated implant for addressing the requirement of a device that supports the aneurysm neck while remaining both extra and intra aneurysmal.

**Figure 4:**
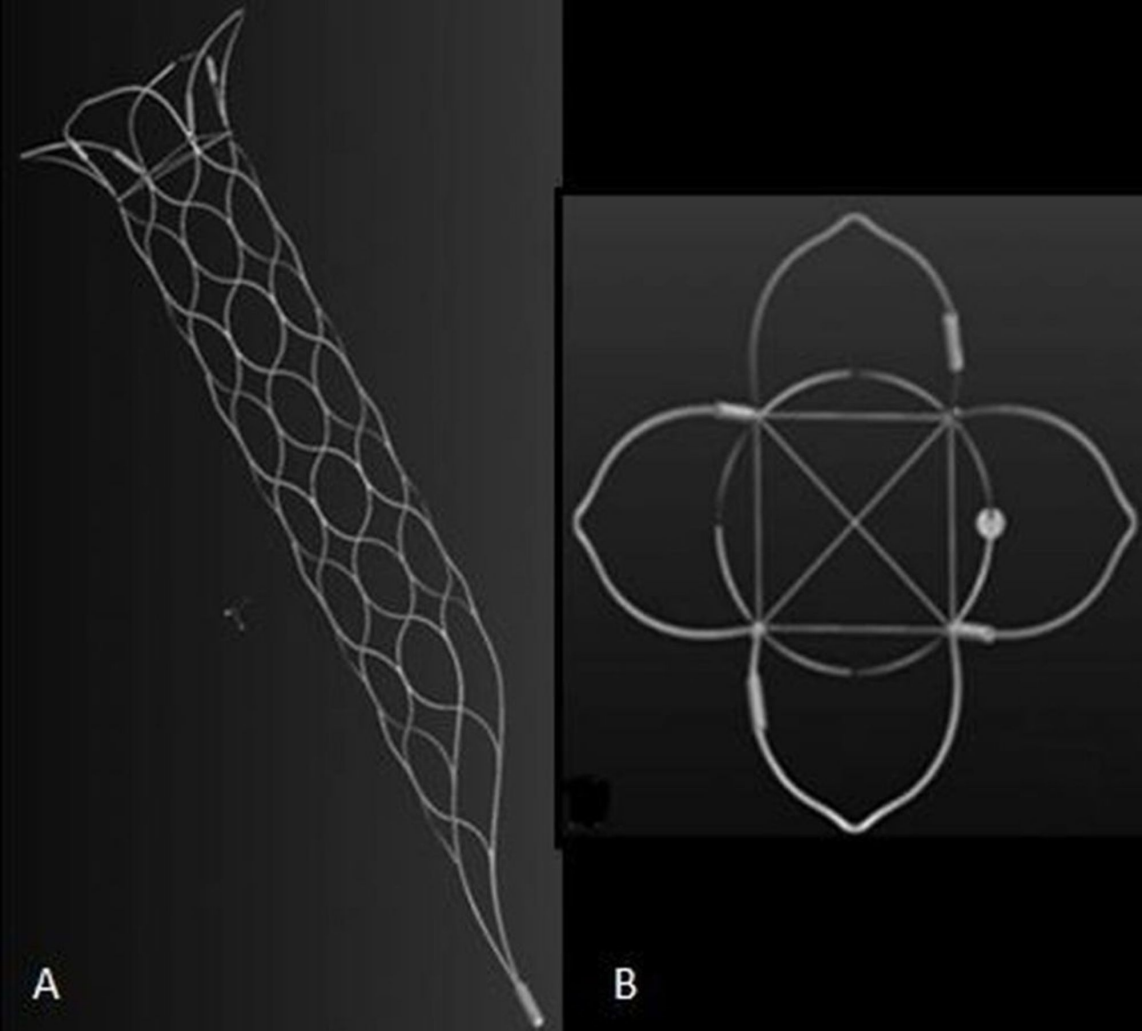
pCONus - a stent-mimicking, nitinol-based laser-cut device

The pCONus is a stent-mimicking, nitinol-based laser-cut device with a radially flared flower-like crown distally that anchors at the aneurysm neck providing a bridging support for coiling. Additional support to the coil mass is provided by six nylon fibers forming a net that creates a mechanical barrier between the aneurysm and the parent vessel (Figure 4).

**Figure 5:**
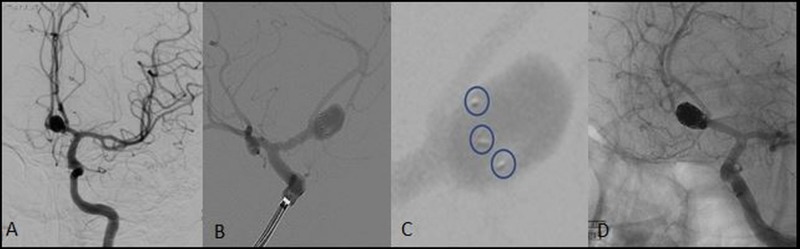
The pCONus device for the treatment of an aneurysm of the anterior communicating artery (AcomA) A: Anterior-posterior (AP) view of the left internal carotid artery (ICA) showing a saccular shaped wide-neck anterior communicating artery (AcomA) aneurysm. B-C: Oblique view of the left ICA showing pCONus in place within the aneurysm. Device markers can be seen as radiopaque dots marked by three circles. The extra-aneurysmal portion of the pCONus is not well visualized. D: Post coiling angiogram shows exclusion of the aneurysm.

Fluoroscopic visualization is made possible by radiopaque markers at the proximal and distal end of the device. The pCONus is electrolytically detachable using available coil-detachment systems. Complete deployment and recoverability of the device is helpful in its optimal placement (Figure 5) [21].

The device has been evaluated in a retrospective study by Perez et al. The study included 28 patients with aneurysms of both anterior and posterior circulation. Insertion and deployment of the pCONus with subsequent coiling was performed in all cases. The study did not report any clinically evident complications, permanent neurologic deficits, or death related to the pCONus deployment [21].

Another retrospective study conducted by Gory et al. in four European centers included only middle cerebral artery aneurysms. No procedural angiographic complications were reported in this study. Reversible neurologic complications were noted in 5% (2/40) and permanent neurologic complications in 2.5% (1/40) at one month. There was no mortality at a mean follow-up of 6.8 months available for 33 aneurysms (82.5%). Stable or improved results were observed in all except three cases [22].

Prospective studies are ongoing for further evaluation and safety of this relatively new device.

PulseRider

**Figure 6:**
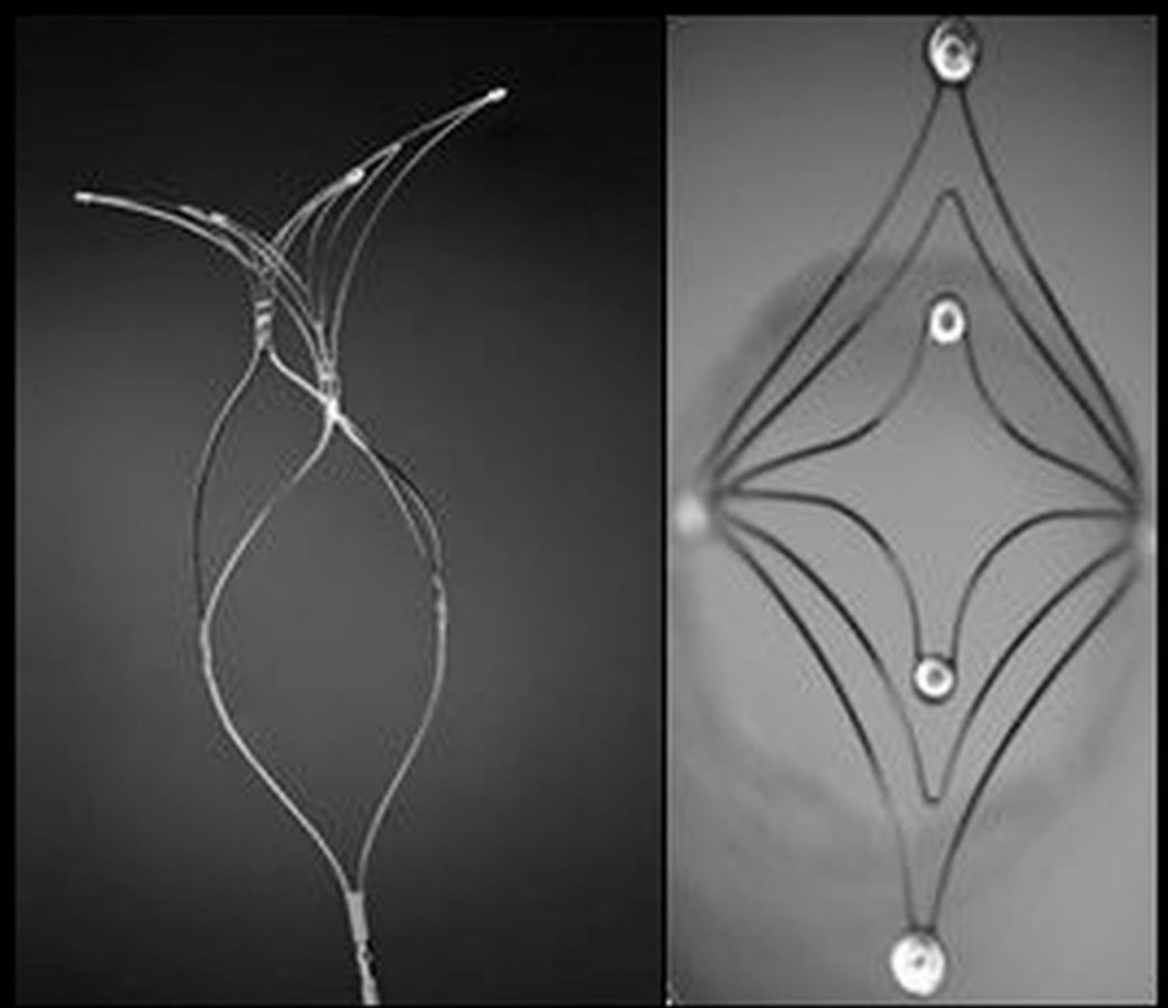
The PulseRider Modified from [24].

The PulseRider is another device for treating aneurysms with asymmetric geometry that evolved in early 2009. It is a self-expanding nitinol implant available in T and Y shapes for conforming to different anatomy. The device presents eight radiopaque markers and is delivered via a stainless-steel wire attached at its base. The delivery wire also has the additional provision of torque, which allows altering the position of the device if required. The device is electrolytically detached through the Pulsar detachment system. The designers of the device claim that the open leaflet structure provides unrestricted access to the microcatheter and entering through the mid markers isn’t a hard and fast rule. Compared to a conventional stent, the PulseRider has less metal and most of the surface area coverage is focused at the aneurysm neck (Figure 6) [23].

The device is being evaluated for its safety and probable benefit for the treatment of wide-neck, bifurcation aneurysms in various trials. The ANSWER (Adjunctive Neurovascular Support of Wide-neck Aneurysm Embolization and Reconstruction) trial in the United States enrolled 34 patients. In all patients, the device was delivered and deployed. Immediate Raymond I or II occlusion was achieved in 82.4% and progressed to 87.9% at the 6-month follow-up. A modified Rankin Score of two or less was seen in 94% of patients at six months [24].

Barrel stent

**Figure 7:**
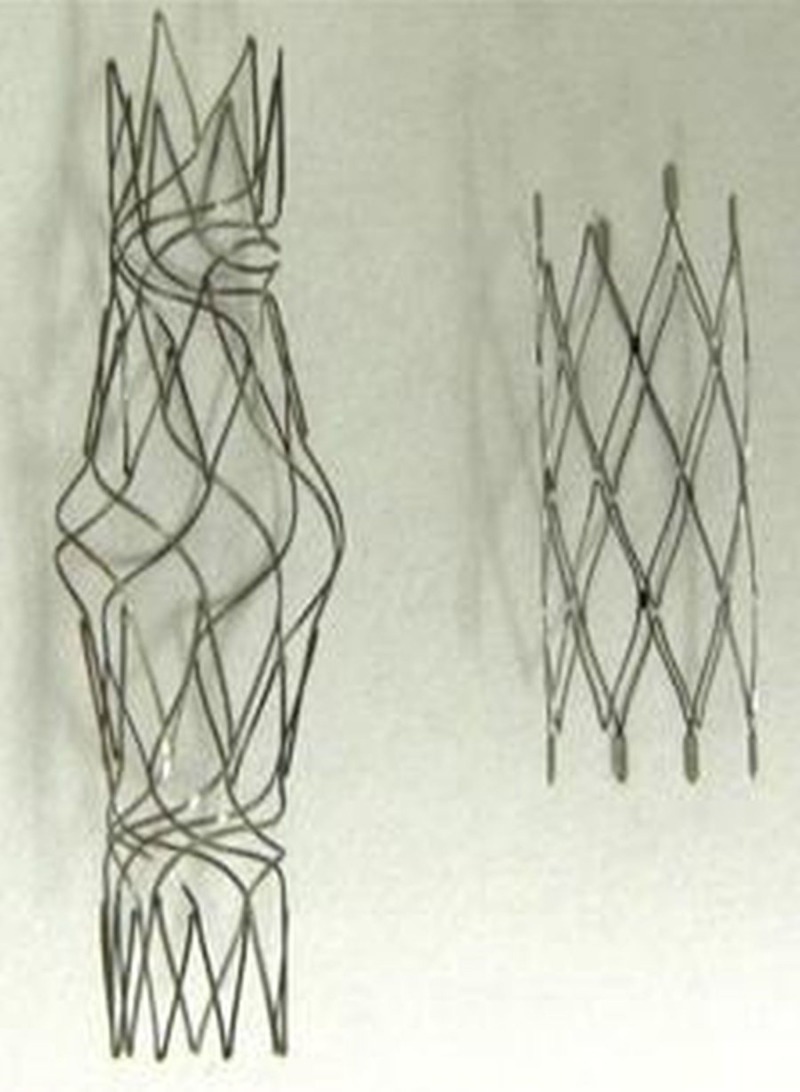
The barrel stent Modified from [25].

Reverse medical has designed a self-expanding stent with a property of mid portion expansion and is called the Barrel stent (Figure 7).

**Figure 8:**
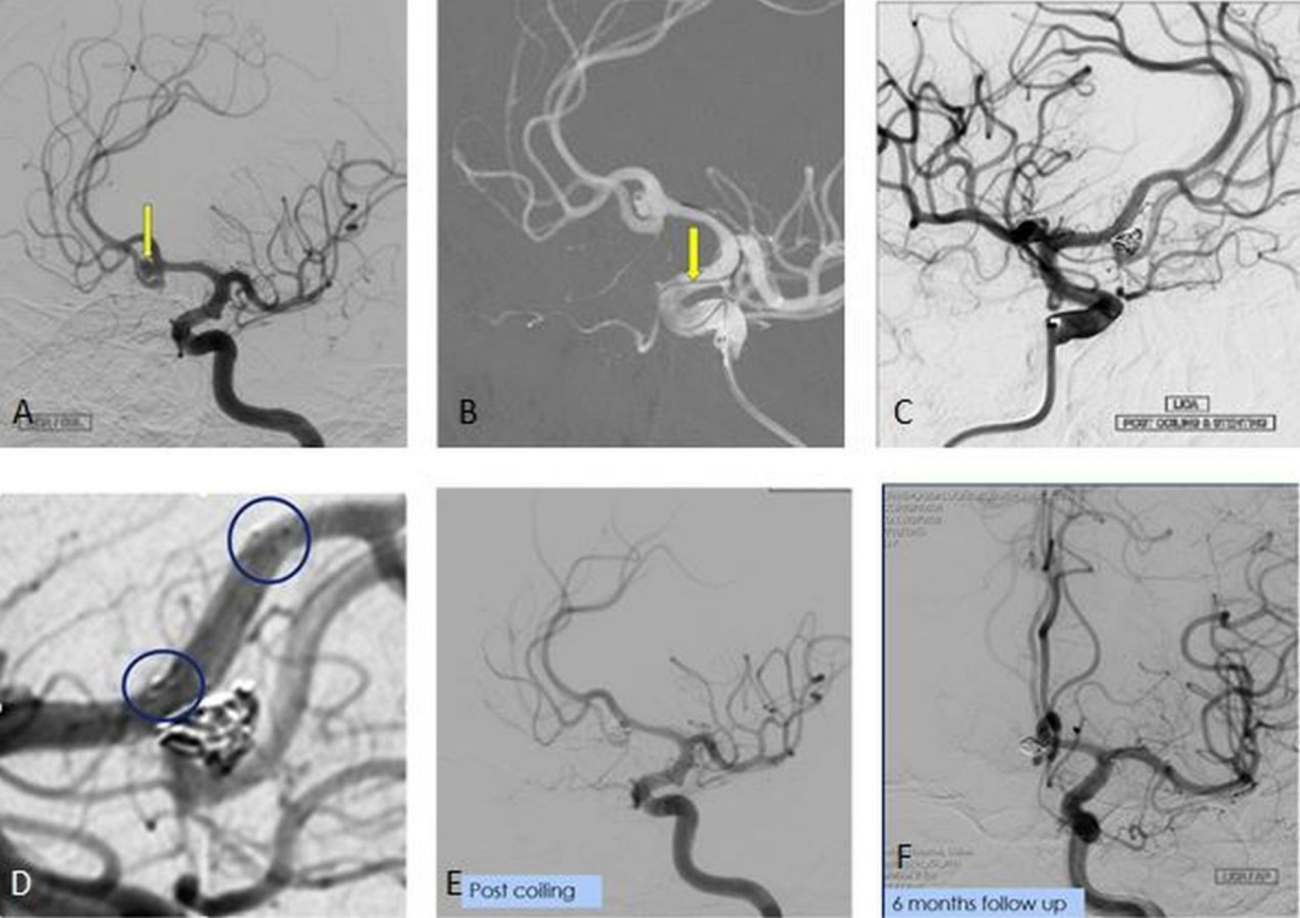
The barrel stent A: Oblique view of the left internal carotid artery (ICA) showing a wide-neck lobulated saccular aneurysm. B: Microcatheter in place (arrow). The tip of the microcatheter can be seen in the aneurysm sac as a black dot. C-D: The barrel stent is in place extending from the A1 to the A2 segment of the anterior communicating artery (AcomA) centered at the aneurysm neck. The stent is marked by circles in D. E: Immediate angiographic result showing exclusion of the aneurysm with the patent parent vessel. F: Six-month follow-up shows stable condition with no evidence of recurrence.

Expansion of the central part recapitulates the concept of assisted coiling using compliant balloons. The significant expansion of the central part maximizes neck coverage in branching locations and facilitates embolization of bifurcation aneurysms (Figure 8) [25].

Treatment by flow diversion

**Figure 9:**
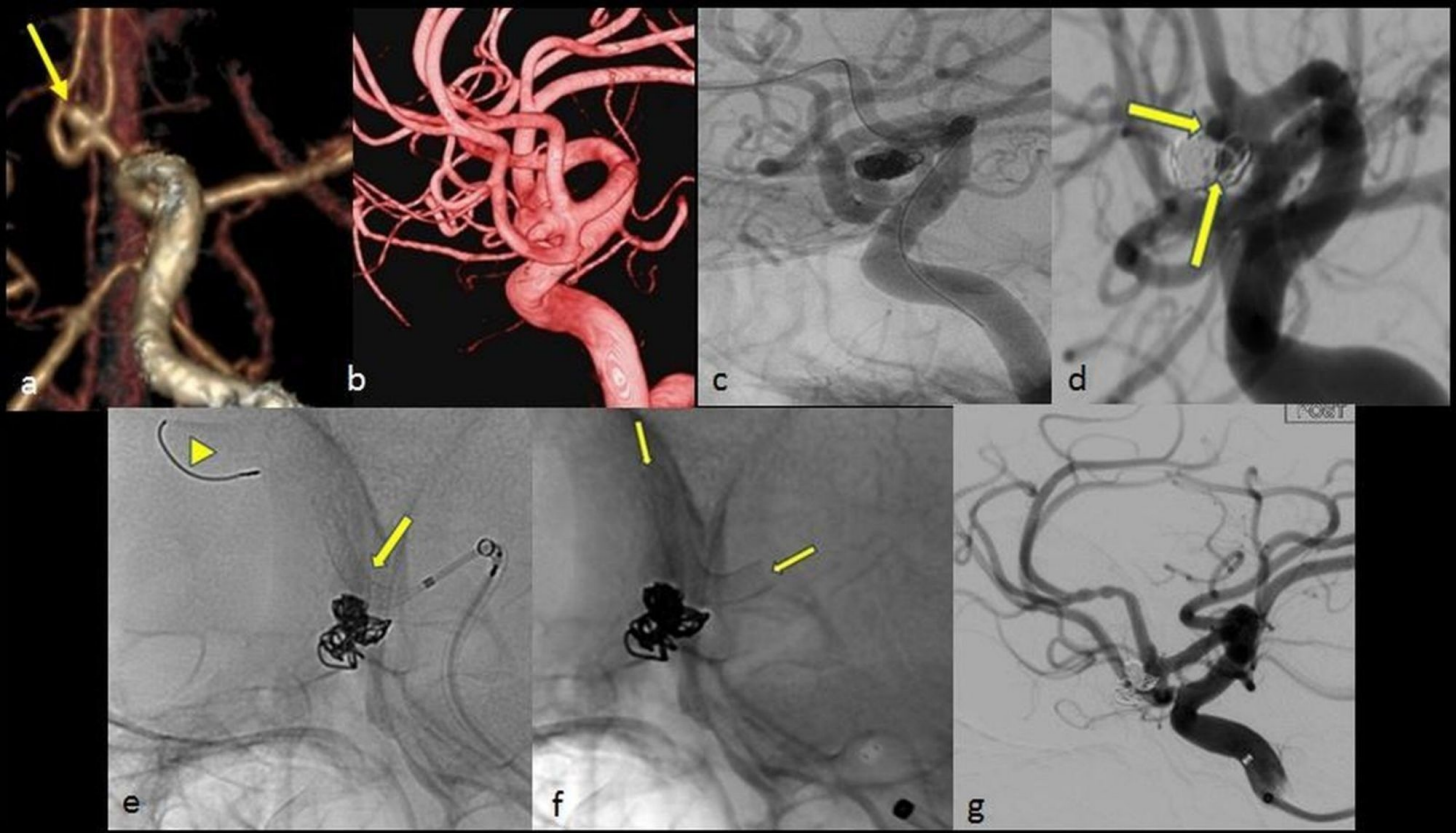
A flow diverter stent for the treatment of an anterior communicating artery (AcomA) aneurysm A: Multiple intensity projection image of the left internal carotid artery (ICA) showing a wide-neck anterior communicating artery (AcomA) aneurysm (arrow). B: 3D angiographic image showing multiple fundal nipples, likely site of rupture in this aneurysm. C: Coiling performed as an emergency procedure to prevent rebleed. D: A seven-month follow-up angiogram shows persistent filling of the aneurysm at its neck (arrows). E-F: A flow diverter (SILK, Balt Extrusion, Montmorency, France) was placed extending from the A1 to the A2 segment of the AcomA across the aneurysm neck. The distal access wire is marked by the arrow head. Flow diverter - arrows in E and F. G: Reduced hemodynamic interchange between the aneurysm and the parent artery with optimal parent artery reconstruction as seen on the final angiogram.

The development of flow diverters was a paradigm shift in aneurysm treatment. These are low-porosity stent-like devices which aim at reducing hemodynamic interchange between the aneurysm and the parent artery, allowing endoluminal remodeling to induce aneurysm thrombosis. The scaffold provided by the flow diverters promotes neointimal growth at the aneurysm-parent artery interface helping further to thrombose the aneurysm. As opposed to the conventional coil embolization techniques, which are meant to target the aneurysm sac and provide exclusion of the aneurysm immediately at the end of procedure, flow diverters cause aneurysms to occlude over time [26]. Food and Drug Administration (FDA) approval of the first flow diverter, i.e. Pipeline device (PED, Medtronic/Covidien, California, USA), occurred following the results of the Pipeline for Uncoilable or Failed Aneurysms (PUFS) trial in 2013 (Figure 9) [27].

Currently several other flow diverters are also available, including Silk (SILK, Balt Extrusion, Montmorency, France), Fred (MicroVention, CA, USA), Surpass (SURPASS, Stryker Neurovascular, CA, USA), and p64 (Phenox GmbH, Bochum, Germany).

Because of being a stent-like design, dual antiplatelet therapy is mandatory prior to implantation of the device. Patients must be loaded with aspirin and clopidogrel prior to the procedure. Most centers use 75-150 mg of aspirin and clopidogrel in 75 mg daily dose post procedure. This regime is continued for a duration of six months. Aspirin is typically continued lifelong while clopidogrel is stopped after six months. Cases of ruptured aneurysms requiring acute treatment create a treatment challenge on account of the above mentioned antiplatelet therapy [28].

The newly designed Pipeline™ Flex Embolization Device (PFED) (Medtronic, MN, USA) with Shield Technology™ utilizes a phosphorylcholine (PC) surface treatment to the implant combined with the PFED delivery system. The Shield Technology™ surface treatment applied to the implant is an inert polymer material created to mimic the outer membrane of a human red blood cell. PC is naturally abundant on the surface of red blood cells. Coating or treating a device surface with a PC-containing polymer results in physiologic mimicry of the cell membrane, which can be the basis of reduced thrombogenicity and possibly reduction in the need for dual antiplatelet therapy [29].

Although a flow-diverter is a novel treatment option, it is associated with complications and its safety is reported to be somewhat inferior to other available aneurysm treatment options. Various studies have reported complications like delayed intra-parenchymal hemorrhage, ischemic stroke, side branch occlusion, infarction related to perforator vessel closure, and delayed rupture of aneurysm [30-32]. The delayed rupture of aneurysms can be explained by the incomplete coverage of the neck with persistent flow into the sac. Vessel perforations have been reported to occur with distal access wire and cases of balloon inflation used for remodeling the implanted device [25].

A meta-analysis conducted by Lv et al. reported an overall technical failure and complication rate of 9.3%, vessel perforation and misplacement in 0.9%, and stent migration and ICA dissection in 0.5% cases [33].

In another meta-analysis by Yao et al., the complete aneurysm occlusion rate was noted to be 84.23%. The procedure-related neurologic mortality was 0.87%, while the procedure-related neurologic morbidity rate was 5.22%. The study found that 1.42% of the cases had intracranial hemorrhage while the ischemic rate was 2.35%. The subarachnoid hemorrhage (SAH) rate was 0.03% while the procedure-related permanent morbidity was 2.41% [34]. These results depict that the use of flow diverters for wide-neck aneurysms is still associated with uncertainties; hence, the use of flow diverters in small-sized or blister-like aneurysms, aneurysms at vessel bifurcations, or cases of dysplastic vessel segments with multiple aneurysms requires further studies to evaluate their efficacy and safety [27].

## Conclusions

Wide-neck intracranial aneurysms are challenging to treat. Although surgical treatment is a viable choice, endovascular management is the preferred treatment option. Endovascular management of wide-neck aneurysms has evolved with the advent of novel devices like highly conformable balloons, intracranial stents, and various stent-like devices as well as improvement in operator skills. Although stent-assisted coiling is a huge step forward in managing these difficult cases, it is mainly indicated for recanalized and unruptured cases, considering the requirement of concomitant anticoagulation to prevent stent thrombosis. The advent of new flow diverters with shield technology is a leap forward in the right direction and seems like a promising step towards circumventing this issue. The development of devices like pCONus, PulseRider, and stents of different architecture has allowed treatment of aneurysms with unfavorable configuration. Although the continuous development of techniques, skills, and devices allows the interventionists of this era to offer a wide range of therapeutic options to patients, controlled clinical trials for these devices and multidisciplinary discussion for each case is necessary to provide the best possible care to every patient.
